# Insights from Tracing the Evolution of Automated Digital Mental Health Interventions in China

**DOI:** 10.31083/AP49906

**Published:** 2026-01-12

**Authors:** Jin Han

**Affiliations:** ^1^Division of Arts and Sciences and Center for Global Health Equity, New York University Shanghai, 200124 Shanghai, China; ^2^Black Dog Institute, University of New South Wales, Sydney, NSW 2031, Australia

Fully automated digital mental health interventions (DMHIs), also known as Type 4 DMHIs, are interventions delivered through technology with minimal human support usually outside of healthcare. These interventions are commonly delivered by websites, mobile-based apps, chatbots, and text-messaging interventions. Although previous study [1] has indicated higher levels of human involvement, e.g., providing guidance or connecting users with others, can enhance user engagement with DMHIs, automated DMHIs are advanced in that they are non-consumable interventions. They can be repeatedly used at low cost, especially when applied on a large scale. Therefore, this type of DMHI could be well-suited for countries like China that have large populations and high mobile and internet penetration rates, while still growing its health infrastructure and personnel.

China accounts for 17% of the global burden of mental, neurological, and substance use disorders [2]. In recent decades, China has undergone significant development in mental health care, including DMHIs. The emergence of generative artificial intelligence (Gen-AI) presents a promising direction for advancing DMHIs by enabling a high level of personalization at scale, and potentially addressing low engagement issues with DMHIs. Since automated DMHIs have shown effectiveness in improving mental wellbeing and mental health symptoms in both general and clinical populations, examining the evolution of automated DMHIs in China at this critical juncture could yield insights, offering implications for other countries facing similar challenges. This analysis outlines automatic DMHI examples as applied to the prevention and treatment of mental disorders. The evolution of automated DMHIs in China over the past decades can be divided into three stages.

## 1. Beginnings: 2010s to 2020s—The West Met China 

Research and implementation of automated DMHIs in China dates back to the early 2010s, trailing behind international efforts by approximately a decade [3]. Most automated DMHIs from this period were either web-based platforms or standalone mobile-based apps, with half of them translated and adapted from English DMHIs for local Chinese use. Typical programs included the Healthy Online Self-help Center (a web-based intervention for internet addiction among college students), the Chinese My Trauma Recovery (a web-based self-guided program for traumatized individuals), the Spirits Healing (a mobile-based mindfulness app for maternal perinatal depression), and the Happy Quit (a text-messaging-based smoking-cessation intervention). Although these programs demonstrated efficacy in reducing mental health symptoms, most of the Chinese DMHIs developed during this period are no longer accessible, despite the continued availability of their English counterparts. That suggests a potential lack of sustainable implementation strategies for automated DMHIs in China during the early stage.

## 2. Two Decades in Three Months: 2020 to 2023—The COVID-19 Accelerator

The coronavirus disease 2019 (COVID-19) pandemic significantly accelerated the growth of telemedicine. A national study in the United States reported a 766% increase in telemedicine encounters during the first three months of the pandemic [4]. A similar trend was observed in China, where monthly online consultations increased nearly fivefold during the pandemic [5]. Although telemedicine itself falls outside the scope of the current analysis because it involves human support, the pandemic also accelerated the development of automated DMHIs in China. This shift was particularly driven by new technologies such as virtual reality (VR), which found a surge in applications in clinical settings. VR-based distraction and mindfulness techniques were quickly adopted to alleviate mental health symptoms, including anxiety and depression, in clinical populations such as pediatric patients [6], cancer patients [7], and Parkinson’s disease patients [8] in China. However, all the identified VR interventions were developed and tested primarily in clinical settings with restricted public availability. The lack of dissemination of automated VR interventions to the general population was suggested to be related to the economic cost of VR technology. Although VR devices became increasingly available over the decade, the cost for individual use remained high. Computer-generated 3D scenes are being explored as a potential cost-effective alternative for public use. Meanwhile, there was a growing trend during this period to broaden the focus beyond mental health issues to address overall health issues, in line with the One Health concept. A notable example is the Lifestyle Hub [9], a smartphone-based multicomponent lifestyle intervention. This eight-week intervention program significantly improved depressive symptoms, anxiety, and stress, and showed a sustained benefit at a one-month follow-up. It is expected to be publicly available soon.

## 3. New Era: 2023 to Present—The Rise of Gen-AI

In December 2022, OpenAI launched its groundbreaking application, ChatGPT. The dialogue-based format of this Gen-AI application lowered the barriers for non-programmers, enabling users to leverage AI for mental health applications. Two years later, the Chinese platform DeepSeek emerged as a strong competitor. Although the concerns that AI would replace clinicians have not yet materialized, there is no doubt that Gen-AI will reshape the automatic DMHIs. A recent article in the New England Journal of Medicine AI reported the first randomized controlled trial of a fully Gen-AI therapy chatbot, Therabot [10]. The intervention demonstrated a significant reduction in depression and anxiety symptoms among clinical groups, in comparison to a waitlist control group. Notably, participants rated the therapeutic alliance during the intervention as comparable to that with human therapists. Although no practical studies on Gen-AI in Chinese mental health were identified in the current search, Chinese mental health professionals have proposed a structured conceptual framework called CORE to assess and mitigate the risks associated with using Gen-AI in mental health prevention and intervention [11].

Upon reviewing progress over the past two decades, it was clear that China has made substantial advancements in using digital technology to prevent and treat mental disorders. There has been a notable acceleration in the country’s practice that aligns with international trends in the field, starting with the adaptation of web-based and mobile-based DMHIs developed overseas for the local context, rapidly adopting emerging digital technologies, transitioning toward system-oriented DMHIs in the wake of COVID-19, and more recently, entering the recent global trends in the use of Gen-AI for DMHIs. However, there are several key areas that warrant further investigation. First, there is an urgent need to expand current digital initiatives to underserved populations. Most studies [12, 13] conducted thus far have focused on young people and adults, with limited evidence and practice involving elderly populations, ethnic minorities, or individuals in economically or socially disadvantaged situations. Reaching these vulnerable groups through the use of automated DMHIs could help address gaps in the current health system in a cost-effective manner. Second, although progress has been made, there remain challenges related to the sustainability and accessibility of automated DMHIs in China. Exploring sustainable funding models and using strategies rooted in implementation science can be helpful to ensure that these innovations remain effective and accessible beyond the trial phase. Third, the rise of Gen-AI is expected to facilitate more personalized prevention and treatment for mental health conditions. This could further support the remote delivery of high-quality mental health services and complement third-wave psychotherapies, which emphasize the importance of considering local contexts when delivering treatment.
